# BK Channel-Mediated Microglial Phagocytosis Alleviates Neurological Deficit After Ischemic Stroke

**DOI:** 10.3389/fncel.2021.683769

**Published:** 2021-07-01

**Authors:** Shuxian Huang, Tingting Chen, Qian Suo, Rubing Shi, Haroon Khan, Yuanyuan Ma, Yaohui Tang, Guo-Yuan Yang, Zhijun Zhang

**Affiliations:** ^1^Shanghai Jiao Tong University Affiliated Sixth People’s Hospital, School of Biomedical Engineering, Shanghai Jiao Tong University, Shanghai, China; ^2^Department of Neurology, Ruijin Hospital, School of Medicine, Shanghai Jiao Tong University, Shanghai, China

**Keywords:** ischemic stroke, BK channels, microglia, phagocytosis, ERK

## Abstract

Microglial phagocytosis benefits neurological recovery after stroke. Large-conductance Ca2^+^-activated K^+^ currents are expressed in activated microglia, and BK channel knockout aggravates cerebral ischemic injury. However, the effect of BK channels on microglial phagocytosis after ischemic stroke remains unknown. Here, we explored whether BK channel activation is beneficial for neurological outcomes through microglial phagocytosis after ischemic stroke. ICR mice after transient middle cerebral artery occlusion (tMCAO) were treated with dimethyl sulfoxide (DMSO), BK channel activator NS19504, and inhibitor Paxilline. The results showed a decrease in BK channel expression after tMCAO. BK channel activator NS19504 alleviates neurological deficit after experimental modeling of tMCAO in mice compared to the control. Furthermore, we treated primary microglia with DMSO, NS19504, and Paxilline after oxygen glucose deprivation (OGD). NS19504 promoted primary microglial phagocytosing fluorescent beads and neuronal debris, which reduced neuronal apoptosis after stroke. These effects could be reversed by BK channel inhibitor Paxilline. Finally, NS19504 increased relative phosphorylated extracellular signal-regulated kinase 1/2 expression compared to the Paxilline group at the third day after stroke. Our findings indicate that microglial BK channels are a potential target for acute stage of ischemic stroke therapy.

## Introduction

Stroke is the major cause of death and long-term neurological disability, seriously threatening quality of life in China and across the world (Wu et al., 2019). Ischemic stroke, comprising 80% of all stroke cases, annually affects about 4 million people in China (Wang et al., 2019). Current therapeutic approaches for ischemic stroke (recombinant tissue plasminogen activator and thrombolysis) are limited by short time window and the risk of hemorrhage transformation (Xiong et al., 2019; Chang et al., 2021). Timely treatment in the acute stage of ischemic stroke could effectively limit infarct volume and rescue neuronal death in the peri-focal region (Chamorro et al., 2020).

Microglial phagocytosis plays an important role in maintaining CNS homeostasis. During mammalian brain development, microglial pruning overproduced synapses and myelin along with neurons (Cobley, 2018). Microglial phagocytosis is required to eliminate myelin debris and protein aggregates in the aging process (Pluvinage et al., 2019; Zhu et al., 2019). However, under pathological conditions, microglial phagocytosis has shown a biphasic role. Microglial abnormal synapse pruning may lead to developmental disorders such as autism (Paolicelli et al., 2011). Knockout of TREM2 in an Alzheimer’s disease (AD) mouse model inhibited microglial phagocytotic effects on removing Aβ plaques and cell debris as well as induced extracellular lipid accumulation and secondary inflammation (Nugent et al., 2020). Depletion of TDP-43 enhanced microglial phagocytosis of amyloid protein in AD mice, while inducing excessive phagocytosis of synapses, consequently aggravating cognitive disorders (Paolicelli et al., 2017). Hence, the effect of microglial phagocytosis on disease progression and retrogression is worth studying.

Ischemic stroke leads to shortage of oxygen and brain nutrients, triggering cell death at the ischemic core (Adav and Sze, 2020; Xu et al., 2020). Timely clearance of apoptotic cells and cellular debris ameliorates ischemic injury and promotes neuroregeneration and tissue repair leading to improved stroke outcomes (Taylor and Sansing, 2013; Zhang et al., 2019). During the acute stage of stroke, microglia engulf granulocytes within 24 h after damage to alleviate apoptosis of neurons (Neumann et al., 2008). Microglial phagocytosis mediated by TREM2 improved neurological outcomes following experimental stroke (Kurisu et al., 2019), indicating the beneficial role of microglial phagocytosis for neuronal survival after ischemic brain injury. However, knockout of phagocytotic protein mer receptor tyrosine kinase (MERTK) and milk fat globule-epidermal growth factor 8 (MFG-E8) depressed microglial phagocytosis, exhibiting a significant reduction in mouse brain atrophy and improvements in neurological behavior (Neher et al., 2013). These studies suggest that microglial phagocytosis plays controversial roles at different stages of ischemic stroke.

BK channels are highly expressed in the central nervous system (Contet et al., 2016). It consists of channel-forming alpha subunits and four accessory beta subunits that can be activated *via* both intracellular calcium and magnesium levels and membrane potential (Wu et al., 2018, in Chinese). The protective effect of activating BK channels has been proven in many disease models (Soltysinska et al., 2014; Dai et al., 2017; Jacobsen et al., 2018). BK channel activator pretreatment limited intestinal ischemia and reperfusion injury *via* an oxidant-dependent mechanism, reduced tumor necrosis factor-α (TNF-α), and protected mucosal permeability (Dai et al., 2017). Activation of BK channels during acute spinal cord injury improved motor functional recovery (Jacobsen et al., 2018). BK channels were demonstrated as a molecular target of several drugs for ischemic stroke therapy including vitamin C (Li L. et al., 2019), chlorpromazine (Li et al., 2014), and Baifuzi (Chi et al., 2010). BK currents were exclusively recorded in activated microglia (Schilling and Eder, 2007). BK channels in microglia played an indispensable role in morphine-induced hyperalgesia (Hayashi et al., 2016); it could be a molecular target of S-ketamine on relieving neuropathic pain (Hayashi et al., 2011). However, the role of microglial BK channels in acute stage of stroke is obscure.

It has been demonstrated that lipopolysaccharide (LPS)-induced macrophage phagocytosis of myelin was inhibited after BK channel blocker Paxilline treatment (Vanheel et al., 2012); microglia are macrophages located in the central nervous system. Thus, we hypothesized that activation of BK channels during the acute stage of ischemic stroke may promote microglial phagocytosis, which improves neurological outcomes after experimental tMCAO.

## Materials and Methods

### Animal Experiments

All animal experiments were conducted in accordance with the guidelines of the Institutional Animal Care and Committee (IACUC) of Shanghai Jiao Tong University, Shanghai, China. Adult male ICR mice, weighting 28–32 g, were kept in a 12-h light/dark cycle with free access to standard food and water. Mice were randomly divided into four groups: (1) sham group; (2) saline plus dimethyl sulfoxide (DMSO) group; (3) Paxilline group; and (4) NS19504 group. Drugs were administered *via* intraperitoneal injection once a day for three consecutive days after tMCAO. The modified neurological severity score (mNSS) was assessed at 1, 3, 7, and 14 days after tMCAO to evaluate neurological function. Hinging wire tests were assessed at 3, 7, and 14 days to evaluate motor function. At day 3 after tMCAO, three mice were sacrificed for RNA and Western blot analysis while the remaining mice were used for immunostaining examination.

### Transient Middle Cerebral Artery Occlusion Model

The transient middle cerebral artery occlusion (tMCAO) model was performed as previously described (Tang et al., 2014). Briefly, after mice were anesthetized with isoflurane (1.5–2%), a midline neck incision was made, and the right common carotid artery (CCA), internal carotid artery (ICA), and external carotid artery (ECA) were isolated carefully. Then, the CCA and ICA were temporarily ligated with 4–0 silk suture. The distal end of ECA was permanently ligated, and a small incision was made for suture insertion. A silicone-coated 6–0 nylon monofilament suture was carefully inserted from the ECA into the ICA to block the MCA. The insertion depth was 9.5 ± 0.5 mm. After 90 min of occlusion, the suture was gently withdrawn for reperfusion. The blood flow of MCA was measured prior to and after the surgery by laser Doppler flowmetry (Moor Instruments, Devon, United Kingdom). About 80% decline in blood flow indicated successful occlusion of the MCA.

### Neurological and Motor Function Assessment

The 14-score mNSS was used to evaluate mouse neurological function, including motor test (six scores), sensory tests (two scores), and bean balance tests (six scores). Higher scores indicate severe neurological deficits. The format of mNSS was described in a previous study (Jiang et al., 2017).

The hinging wire test was used to investigate the motor function, especially forelimbs, of mice. Mice were placed at the middle of a horizontal wire about 20 cm above the table, and only their forelimbs were allowed to grab the wire at the beginning. The initial score was kept as 10 points; one point was given when mice arrived at either end, while one point was deducted when mice fall off. We recorded the time and score every time mice fall down or arrive at the end during 180 s. We ended up with a graph of scores per second.

### Antagonist and Activator Administration

The selective BK channel antagonist Paxilline (Zhou and Lingle, 2014) and activator NS19504 (Nausch et al., 2014) powder (Alomone Labs, Jerusalem, Israel) were, respectively, dissolved in DMSO to the final storage concentrations of 2 and 50 mM. Mice received a single dose of 14 μg/kg Paxilline or 14 mg/kg NS19504 *via* intraperitoneal injection. The injection concentrations of Paxilline and NS19504 were 500 μM and 5 mM, respectively, having the same concentration of DMSO [10% (Li and Zhao, 2007)]. One μM Paxilline and 10 μM NS19504 were added into primary microglial culture *in vitro*.

### Frozen Section of Mouse Brain

Mice were sacrificed and then transcardially perfused with phosphate-buffered saline (PBS), followed by 4% paraformaldehyde (PFA). Then, the brain was carefully removed from the endocast and rapidly put in isopentane (chilling at −80°C) for 1 min. Finally, the brain was taken out and wrapped in tin foil, stored at −80°C. Before slicing, frozen brain, metal pallet, and blades were placed in the freezing microtome (Leica CM1860, Leica, Nussloch, Germany) at −20°C for 20 min, then the brain was fixed in an optimal cutting temperature compound (OCT, Leica, Nussloch, Germany) on the metal pallet. After OCT solidification, the brain was sliced into 20 μm and the slices were stored at −80°C.

### Western Blotting Analysis

Mice were sacrificed and then transcardially perfused with PBS. The striatum of stroke and the contralateral hemispheres were lysed in extraction buffer, which comprised the following components: 10× radioimmunoprecipitation lysis buffer (RIPA, EMD Millipore Corp., Billerica, MA, United States); 10× phosphatase inhibitor PhoSTOP (Merck KGaA, Darmstadt, Germany); 100× protease inhibitor cocktail (Beyotime, Shanghai, China); 100× phenylmethanesulfonyl fluoride (PMSF, Thermo Scientific, Waltham, United Kingdom); and ddH2O = 10:10:1:1:78. After ultrasonic homogenization, samples were centrifuged at 12,000 rpm (4°C) for 20 min, and a liquid supernatant was taken for subsequent steps. The concentration of each protein sample was determined using the BCA kit (Thermo Scientific, Waltham, United Kingdom), and each amount of protein (30 μg per line) was loaded for SDS-PAGE. Then, proteins were transferred to polyvinylidene fluoride membrane (Merck KGaA, Darmstadt, Germany). Membranes were blocked with 5% non-fat milk for 1 h and incubated with the primary antibodies of BK channels (1:1000 dilution, Abcam, Cambridge, United States), p-ERK (1:1000 dilution, CST, Massachusetts, United States), total-ERK (1:1000 dilution, CST), and β-actin (1:1000 dilution, Invitrogen, Carlsbad, CA, United States) for more than 16 h at 4°C. After washing three times with TBST buffer, membranes were incubated with a horseradish peroxidase (HRP)-conjugated secondary antibody for 1 h at room temperature. Protein bands were detected with a chemiluminescent HRP substrate (MeilunBio, Dalian, China). The results were analyzed by ImageJ software.

### Real-Time PCR

Mice were sacrificed and then transcardially perfused with PBS. Total RNA was isolated from the striatum of the infarcted area using TRIzol reagent (Invitrogen, Carlsbad, CA, United States). After reverse transcription, quantitative real-time PCR was performed using primers specific for the genes encoding BK channels and inflammatory mediators. All procedures were performed following the manufacturer’s protocol (Yisheng, Shanghai, China).

### Sequence of Primers Used in This Study

**Table:** 

Gene name	Forward primer (5′–3′)	Reverse primer (5′–3′)
BK	GCGGCTTGAAGATGAGCAG	TGCCAGGAATTAACAAGGGGT
IL-1α	TCGGCAAAGAAATCAAGATG	ATGGTCAATGGCAGAACTGTAG
IL-1β	TACATCAGCACCTCACAAGC	AGAAACAGTCCAGCCCATACT
IL-6	TGATGCACTTGCAGAAAACAA	GGTCTTGGTCCTTAGCCACTC
IL-10	GCGCTGTCATCGATTTCTCCC	TGGCCTTGTAGACACCTTGG
TNF-α	ACCCTCACACTCAGATCATCTT	GGTTGTCTTTGAGATCCATGC
TGF-β	CACCGGAGAGCCCTGGATA	TGTACAGCTGCCGCACACA
GAPDH	AAATGGTGAAGGTCGGTGTG	AGGTCAATGAAGGGGTCGTT

### Immunofluorescent Staining

Brain sections were fixed with 100% methanol (chilled at −20°C) for 2 min and incubated with 0.3% Triton X-100 solution for 10 min, then subjected to sodium citrate buffer microwave antigen retrieval and blocked with 1% bovine serum albumin (BSA) for 1 h. Then, sections were incubated with primary antibodies anti-BK α-subunit (1:50, Santa Cruz, TX, United States), GFAP (1:200, EMD Millipore Corp., Billerica MA, United States), IBA1 (1:200, Novusbio, Littleton, CO, United States), CD11b (1:200, Bio-Rad, Hercules, CA, United States), NeuN (1:200, EMD Millipore Corp., Billerica, MA, United States), and MAP2 (1:200, EMD Millipore Corp., Billerica, MA, United States) overnight at 4°C. After washing three times with PBS, sections were incubated with fluorescent conjugated secondary antibodies for 1 h at 37°C. Sections were washed again three times with PBS and air dried in a dark room, then covered by an anti-fluorescence attenuation sealant (with DAPI, Meilun, Dalian, China) and coverslip. The fluorescent images were taken by confocal microscope (Leica, Solms, Germany).

Cell coverslips were carefully taken out and put in a 24-well plate, and washed by PBS, then treated the same as the brain sections. Antigen retrieval was not needed for the cell slide staining.

### Cell Culture

Primary microglia and neurons were isolated from cerebral cortices of Sprague Dawley rats (SD, JSJ, Shanghai, China) born within 24 h. Briefly, for primary microglia, isolated cells were plated into poly-D-lysine (PDL, Sigma-Aldrich, St. Louis, MO, United States)-coated 75-cm^2^ flasks at the density of 1 × 10^5^–1.4 × 10^5^ cells/cm^2^. Cells were cultured in Dulbecco’s modified Eagle medium (DMEM, Gibco Laboratories, Grand Island, NY, United States) with 10% fetal bovine serum (FBS, Gibco Laboratories, Grand Island, NY, United States). Microglia were collected by shaking flasks in ∼10 days after seeding then plated into a 24-well plate and cultured for 24 h for phagocytosis assay and coculture.

For neurons, isolated cells were plated into six-well plates coated with PDL at the density of 6 × 10^5^–8 × 10^5^ cells/well and cultured in DMEM with 10% FBS. After 4 h, the medium was changed to Neurobasal medium (Gibco, Carlsbad, CA, United States) with B27 (Gibco, Carlsbad, CA, United States). Neurons were ready to use in ∼5 days after seeding. All cells were kept in a humidified incubator containing 5% CO_2_/95% O_2_ at 37°C.

### CCK8 Assay

Microglia were seeded in a 96-well plate at a density of 2 × 10^5^ cells/well with 100 μl conditional media, respectively, containing 0.1, 1, 2, 5, and 10 μM Paxilline and 1, 5, 10, and 100 μM NS19504. Five duplicate wells were set for each concentration. After a 12-h treatment, 10 μl CCK8 was added into each well. The plates were incubated for 1–4 h in the incubator. The absorbance at 450 nm was measured by a microplate reader.

### Oxygen-Glucose Deprivation (OGD) and Reoxygenation Model

Microglia were seeded in 24-well plates at a density of 15 × 10^5^–20 × 10^5^ cells/well with 500 μl conditional media. Cells were washed twice with sterile water and once with PBS, then cultured in glucose-free DMEM. Cultures were placed into a round anaerobic chamber filled with 95% N_2_ and 5% CO_2_ and kept at 37°C for 1 h of oxygen-glucose deprivation (OGD). An oxygen meter was placed in the chamber to monitor the oxygen concentration. Then, the medium was replaced by DMEM with 10% FBS. Cells were maintained under normoxic conditions for 11 h at 37°C for reoxygenation. Neurons were subjected to OGD for 8 h and reoxygenation for 12 h to get debris.

### Phagocytosis Assay

The phagocytosis activity of microglia was assessed by measuring the uptake of fluorescent beads or neuronal debris under a confocal microscope (Leica, Solms, Germany). Fluorescent latex beads (diameter 0.7 μm, CellMeter, United States) showed enhanced red fluorescence when phagocytosed by microglia. After 8 h of OGD and 12 h of reoxygenation, neuron media were centrifuged at 10,000 g for 5 min, resuspended in DMEM twice to obtain neuronal debris. Debris then were stained using the PKH26 kit (Merck, Kenilworth, United States). After OGD, reoxygenation, and 12 h treatment with normal microglia medium containing 1 μM Paxilline or 10 μM NS19504, microglia were incubated in normal medium with beads or debris for 2 h. Cultures were washed with PBS about five times and fixed with 4% PFA. All experiments were repeated at least three times independently, and each experimental group had three parallel wells. Three areas per well were photographed and statistically analyzed.

### Statistical Analysis

Results were presented as the mean ± SEM, and the differences of two groups were analyzed by Students’ *t*-test, three groups analyzed by one-way ANOVA followed by the Bonferroni corrections. A value of *p* < 0.05 was taken as statistically significant. These analyses were done using GraphPad 5.0 software.

## Results

### BK Channel Expression Decreased After tMCAO

**FIGURE 1:**
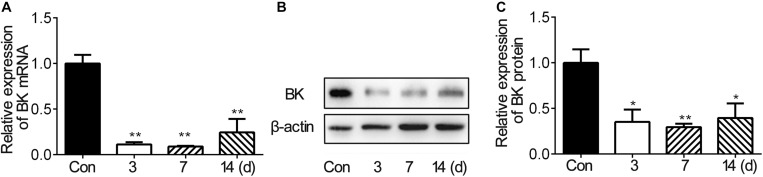
BK expression in the ipsilateral hemisphere decreased after mouse tMCAO. **(A)** Bar graph shows the BK mRNA expression in the mouse ipsilateral hemisphere at 3, 7, and 14 days after 90-min tMCAO. Data are mean ± SE, *n* = 3–7 per group. **p < 0.05. tMCAO vs. control. **(B)** Representative image of Western blot shows BK expression in the mouse ipsilateral hemisphere at 3, 7, and 14 days after 90-min tMCAO. **(C)** Bar graph shows that the semiquantitative data of BK protein levels at 3, 7, and 14 days after tMCAO. Data are mean ± SE, *n* = 3 per group. *p < 0.05, **p < 0.01, tMCAO vs. control.

To investigate the expression of BK channels after stroke *in vivo*, we examined the mRNA and protein levels of BK channels in the peri-infarct area of mice brain after tMCAO. The RNA level of BK channels decreased during 3–14 days after tMCAO (Figure 1A). The protein level of BK channels also reduced after tMCAO and reached the bottom at 7 dpi (Figures 1B,C). These results suggested that BK channels were downregulated in the brain after ischemic stroke.

### BK Channels Primarily Located in Microglia and Neurons

**FIGURE 2:**
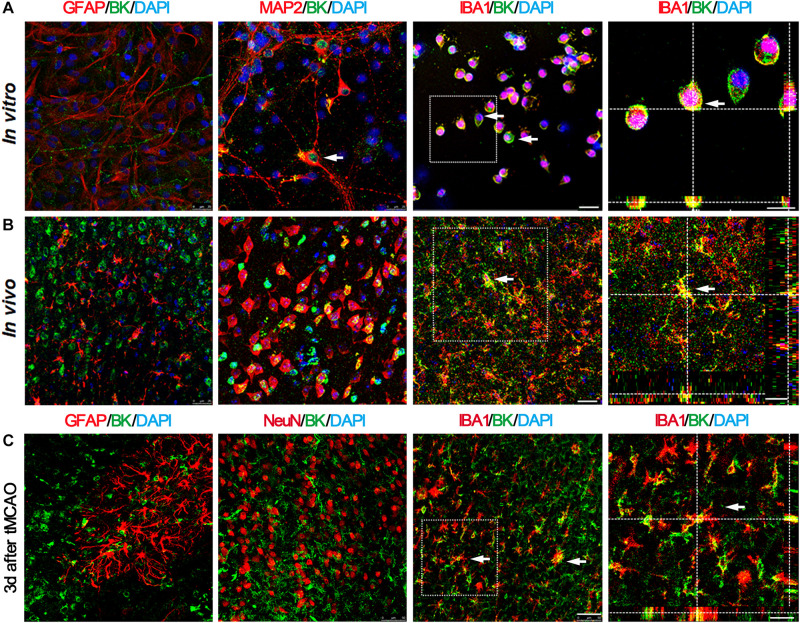
BK channels are mainly expressed in microglia and neurons, but not in astrocytes. **(A)** Representative photomicrographs show BK (green), GFAP (red), MAP2 (red), and IBA1 (red) double staining in primary cultured cells. Scale bar = 25 μm. Right-side image indicates that BK is expressed in the IBA1^+^ microglia by 3D two-photo microscope image. Scale bar = 10 μm. **(B)** Representative photomicrographs show BK (green), GFAP (red), MAP2 (red), and IBA1 (red) double staining in the cortex of the normal mouse brain. Scale bar = 25 μm. Right-side image indicates that BK was expressed within the IBA1^+^ microglia, scale bar = 30 μm. **(C)** Representative photomicrographs of BK/GFAP, BK/NeuN, and BK/IBA1 double staining at 3 days after tMCAO. Scale bar = 50 μm. Right-side image indicated that BK was expressed in the IBA1^+^ microglia by 3D two-photo microscope image. Scale bar = 20 μm.

In order to determine which kind of cell types expressed BK channels after stroke, we sacrificed mice at 3 days after tMCAO and performed immunofluorescent staining. The results showed that BK channels were mainly expressed in microglia and neurons, not in astrocytes (Figure 2A). Similar results were observed *in vitro* (Figure 2B). At 3 days after tMCAO, we found that most BK channels were located in microglia, while few with neurons and astrocytes (Figure 2C), which suggested that BK channels on microglia play a major role after stroke.

### NS19504 Treatment Promoted the Phagocytosis of Primary Microglia After OGD

**FIGURE 3:**
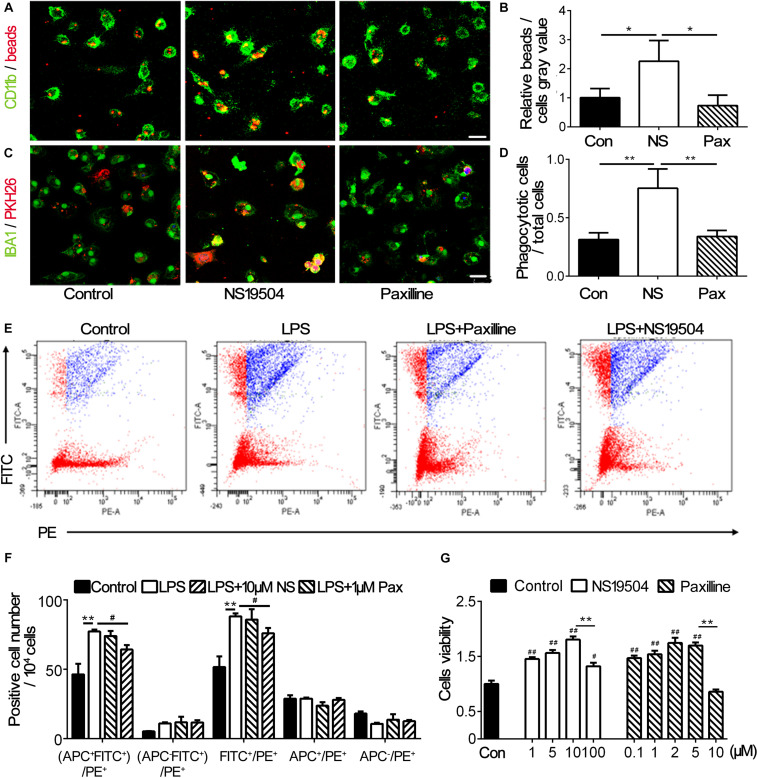
BK channel activator NS19504 promoted microglial phagocytosis after OGD in primary culture**. (A)** Representative images of beads (red) phagocytosed by CD11b^+^ microglia (green) after 1 h of OGD. Control group: primary microglia cultured with basic microglia medium; NS19504 group: primary microglia cultured with 10 μM NS19504 in basic microglia medium; Paxilline group: primary microglia cultured with 1 μM Paxilline in basic microglia medium. Scale bar = 25 μm. **(B)** Bar graph showed that semiquantitative data of the relative bead density versus microglia. Data are mean + SE, *n* = 3 per group. *p < 0.05, control vs. NS19504 or Paxilline vs. NS19504 group. **(C)** Representative photomicrographs showed that neuronal debris (red) phagocytosed by microglia (green) in the NS19504- and Paxilline-treated mice following tMCAO. Scale bar = 25 μm. **(D)** Bar graph shows semiquantitative data of the proportion of microglia that phagocytose neuronal debris. Data are mean + SE, *n* = 3 per group. *p < 0.05, NS19504 vs. control or Paxilline vs. NS19504 group. **(E)** Representative photomicrographs show FITC and PE-positive cells. FITC-labeled beads were phagocytosed by PE-labeled microglia. Primary microglia were divided into the control group (basic microglia medium); LPS group (200 ng/ml LPS); LPS + NS19504 group (200 ng/ml LPS and 10 μM NS19504); and LPS + Paxilline group (200 ng/ml LPS and 1 μM Paxilline). **(F)** Bar graph shows semiquantitative analysis of the proportion of microglia that phagocytose beads. Data are mean + SE, *n* = 3 per group. **p < 0.01, compared with the control group. ^#^*p* < 0.05, compared with the Paxilline group. **(G)** Bar graph shows the result of CCK-8 assay in the primary microglia without or with different concentrations of Paxilline and NS19504 for 12 h of OGD. Data are mean ± SE, *n* = 3 per group. ^#^*p* < 0.05, ^##^*p* < 0.01, compared with the control group. *p < 0.05, **p < 0.01; NS represents for NS19504, and Pax represents for Paxilline.

Previous study has indicated that inhibiting BK channels could block LPS-induced phagocytosis of macrophages (Vanheel et al., 2012). To investigate the effect of BK channel activator NS19504 on microglial phagocytosis, we cultured primary microglia and performed OGD to mimic the condition of stroke *in vivo.* First, we examined the effects of NS19504 and Paxilline concentration gradient on cell viability to determine the optimum dosing concentration. The results showed that 1 μM Paxilline and 10 μM NS19504 were not cytotoxic within effective concentrations (Figure 3G). After OGD and reoxygenation, microglia were treated with NS19504 or Paxilline for 12 h. Then, microglia were incubated with fluorescent beads, which are PH sensitive, so the fluorescence would be enhanced after being phagocytosed. We measured the gray value of fluorescent beads versus CD11b^+^ microglia. The NS19504 group showed increased the relative beads/microglia gray value compared to the control group (Figures 3A,B). Paxilline treatment significantly reduced microglial phagocytosis after OGD compared with the NS19504 treatment. In order to mimic the condition *in vivo*, we substituted neuronal debris for fluorescent beads. After the same treatment, microglia of three groups were incubated with neuronal debris which were stained with PKH26. The results showed that more cells in the NS19504 group engulfed neuronal debris, while microglia of the Paxilline group showed a similar level of phagocytosis with the control group (Figures 3C,D).

To further examine microglial phagocytosis after Paxilline and NS19504 treatment, we also used flow cytometry. We detected PE-labeled CX3CR1^+^ microglia engulfing FITC-labeled beads. After LPS stimulation, microglia were treated with Paxilline or NS19504 or normal microglial medium. The results showed that the LPS group had a higher percentage of PE^+^FITC^+^ cells compared to other groups. Paxilline significantly inhibited LPS-induced microglial phagocytosis (Figures 3E,F).

Since microglial M2 polarization could promote clearance of apoptotic cells and improve tissue repair after ischemic stroke (Yang et al., 2017; Wen et al., 2020), we explored whether stimulation of BK channels could change the polarization of microglia. APC-CD206 was used to label M2 microglia; flow cytometry was used to determine the number of M2 microglia of different groups. Figure 3F shows that the four groups of APC+ cell number had no significant difference, demonstrating that regulation of BK channels had no effect on the polarization of M2 microglia.

### Activation of BK Channels Reduced Neuronal Apoptosis After tMCAO

**FIGURE 4:**
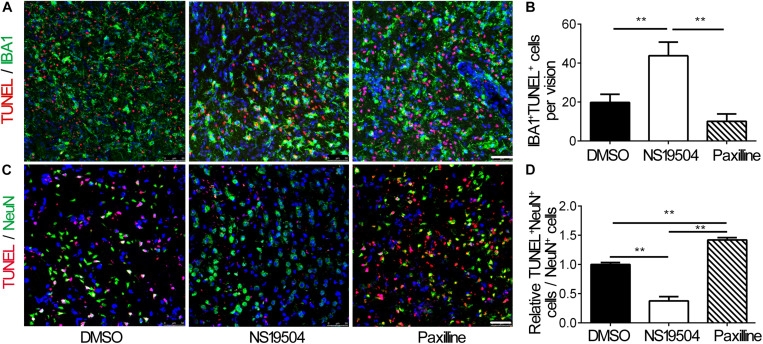
Activation of BK channels promoted the phagocytic function of microglia after tMCAO and decreased neuronal apoptosis**. (A)** Representative photomicrograms of IBA1^+^ microglia (green) and TUNEL^+^ apoptotic cells (red) in the peri-infarct region of brain slice in the DMSO, NS19504, and Paxilline groups at 3 days after tMCAO. Scale bar = 50 μm. **(B)** Bar graph shows quantitative analysis of the number of IBA1^+^ TUNEL^+^ cells per field. Data are mean + SE, *n* = 3 per group. *vs. DMSO group, **p < 0.01. **(C)** Representative photomicrograms of NeuN^+^ neurons (green) and TUNEL^+^ apoptotic cells (red) in the peri-infarct region of brain slice in the DMSO, NS19504, and Paxilline groups at 3 days after tMCAO. Scale bar = 50 μm. **(D)** Bar graph shows quantitative analysis of the number of NeuN^+^/TUNEL^+^ cells per vision. Data are mean + SE, *n* = 3 per group. *vs. DMSO group, **p < 0.01.

Next, we examined the effect of microglial phagocytosis on apoptotic cells after BK channel activation in ischemic mice. We quantified the number of DAPI^+^/Iba1^+^ microglial phagocytosis TUNEL-positive cells, which represented that microglia phagocytes apoptotic cells. The number of phagocytic microglia increased in the NS19504 group compared to DMSO and Paxilline groups, which indicated that microglia of the NS19504 group displayed higher phagocytic activity (Figures 4A,B).

Then, we performed immunostaining to measure the number of apoptotic neurons. Results showed that the number of NeuN^+^/TUNEL^+^ neurons decreased in the NS19504 group compared to the DMSO and Paxilline groups (Figures 4C,D).

### BK Channels Activation Promoted Mouse Outcomes After Ischemic Stroke

**FIGURE 5:**
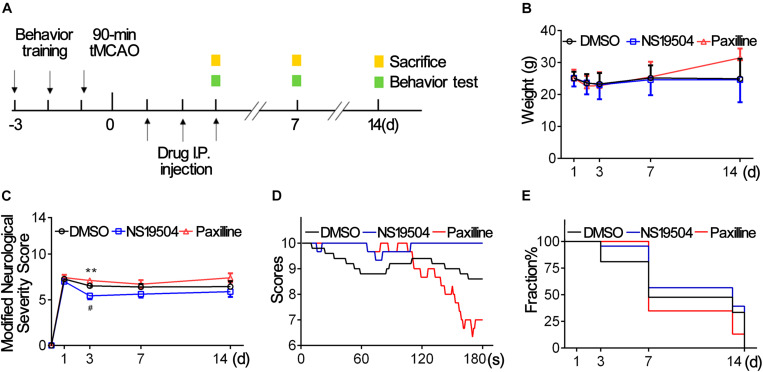
Activation of BK channels ameliorated stroke outcomes. **(A)** Experiment design of the animal experiment. Three days before tMCAO, mice were subjected behavior training. After a 90-min tMCAO surgery, mice were divided randomly into four groups (sham, DMSO, NS19504, and Paxilline). At 1, 3, 7, and 14 days, behavior tests were performed, and protein and RNA samples were collected. **(B)** Weight changes of mice in three groups at 1, 2, 3, 7, and 14 days after tMCAO. **(C)** Modified Neurological Severity Score of three groups at 1, 3, 7, and 14 days after tMCAO. **(D)** Average score per second of three groups of hinging wire at 3 days after tMCAO. **(E)** Survivorship curve during 14 days of three groups after tMCAO. Data are mean + SE, *n* = 21–23 per group at 1 day, *n* = 17–23 per group at 3 days, *n* = 8–13 per group at 7 days, *n* = 3–9 per group at 14 days. *NS19504 vs. Paxilline group, **p < 0.01. ^#^NS19504 vs. DMSO, ^#^*p* < 0.05.

BK channel-knocked out mice show a larger infarct area after ischemic stroke (Liao et al., 2010). To confirm the effect of BK channel activation on stroke outcomes, we treated mice with a BK channel-specific activator (NS19504) or inhibitor (Paxilline) for 3 days after tMCAO. Figure 5A shows the timeline of animal experiments. First, we recorded weight changes of three groups. As shown in Figure 5B, mice lost the most weight at 3 days and there was no significant difference among groups during 14 days after tMCAO. Then, we investigated the number of surviving mice and found that about more than 80% of mice of the Paxilline group failed to live at 14 dpi (Figure 5E), which suggests that inhibition of BK channels at the acute phase affected the survival rate after stroke.

We used mNSS to evaluate the neurological deficit at 1, 3, 7, and 14 days after tMCAO. Hinging wire tests were conducted at 3 days to test motor function. The results showed that the neurological score was lower at 3 dpi in the NS19504 group than in the DMSO and Paxilline groups (Figure 5C). Results of the hinging wire test demonstrated that the score of the NS19504 group was higher compared to that of the DMSO and Paxilline groups (Figure 5D). The Paxilline group gained the lowest end score at 180 s among three groups, which suggests that inhibition of BK channels aggravates motor function deficit. These results demonstrate that activating BK channels could promote mouse motor functions after ischemic stroke.

### Activation of BK Channels Promoted ERK Expression After tMCAO

**FIGURE 6:**
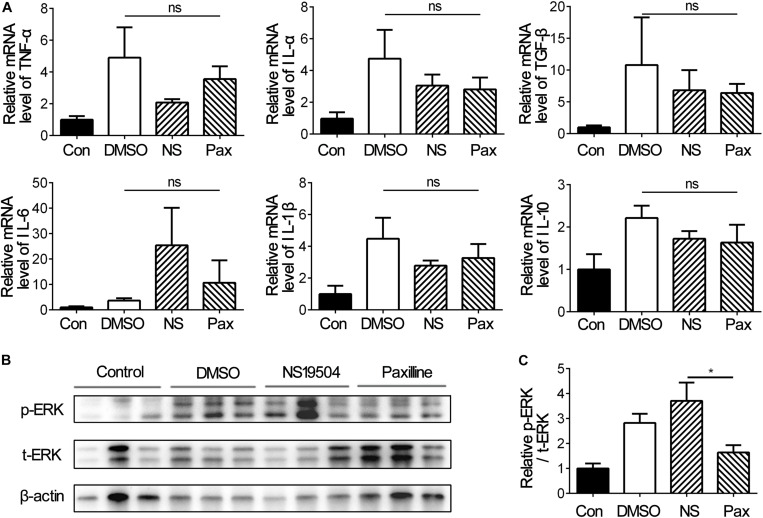
Inflammatory factors and phosphorylated-ERK expressions under different treatments at 3 days after tMCAO**. (A)** Bar graph represents the relative mRNA levels of inflammatory factors including TNF-α, TGF-β, IL-10, IL-6, IL-1β, and IL-1α. Data are mean + SE, *n* = 3 per group. **(B)** Representative image of Western blot of phosphorylated-ERK1/2 (p-ERK), total-ERK1/2 (t-ERK), and β-actin at 3 days after tMCAO. **(C)** Bar graph showing the relative protein levels of *p*-ERK vs. t-ERK at 3 day after tMCAO. Data are mean + SE, *n* = 9 per group. *NS19504 vs. Paxilline group, *p < 0.05; ns, no significance.

Inflammation response is a general feature in nervous system diseases and is the initial defense mechanism against injury (Chung et al., 2019; Li K. et al., 2019; Sarkar et al., 2019). Since NS19504 treatment promoted the outcome of stroke, we detected whether inflammation affects the significant difference between groups, and the mRNA levels of inflammation factors TNF-α, IL-1β, IL-6, IL-10, and TGF-β were examined. The results of RT-PCR showed that there was no difference between three groups, but the Paxilline group had the trend to severer inflammation (Figure 6A).

ERK has been reported to play an important role in TREM2-mediated microglial phagocytosis (Fu et al., 2014) and participate in the regulation of WNK1 to BK channels (Liu et al., 2015). We investigated that ERK1/2 function in BK channels mediated microglial phagocytosis. Western blot was used to detect phosphorylated ERK expression versus total ERK expression at 3 days after tMCAO. The results showed that activation of BK channels upregulated the pERK1/2/tERK1/2 level compared with Paxilline, which suggested that ERK was involved in BK channel-mediated microglial phagocytosis in the early stage of ischemic stroke (Figures 6B,C).

## Discussion

In this study, we explored the role of BK channels in microglial phagocytosis after tMCAO and demonstrated that activation of BK channels during the first 3 days after tMCAO promoted microglial phagocytosis and alleviated neurological deficit after ischemic stroke; ERK was involved in the activation of BK channel-mediated microglial phagocytosis.

Previous studies suggest that BK channels have neurovascular protection in cerebrovascular diseases. Knockout of the BK channel shows more severe behavioral defects and higher mortality (Liao et al., 2010). However, the expression pattern and the mechanism of neuroprotection after ischemic stroke of BK channels are still unclear. In this study, our results showed that the BK channel expression of the mouse ipsilateral hemisphere was continuously lower than that of the sham group from 1 to 14 days after ischemic stroke; BK channels were mainly expressed on microglia and neurons, but almost not expressed on astrocytes in the sham group. At 3 days after ischemic stroke, BK channels are mainly located in microglia but few in neurons. Therefore, the BK channel on microglia may play a key role in post-stroke injury.

Then we used drugs that can efficiently and specifically regulate BK channels, including activators and inhibitors. NS19504 is a novel BK channel activator having a higher specificity than the commonly used BK channel activator NS1619, NS19504, which does not activate the small-conductance potassium channel (IK channels) while activating the BK channels (Nausch et al., 2014; Catacuzzeno et al., 2015). Paxilline is a classic BK channel inhibitor, which is the most effective and selective non-endogenous inhibitor of BK channels (Yu et al., 2016). Besides, NS19504 and Paxilline can both cross the blood–brain barrier. In this study, we found that NS19504 has a protective effect against ischemic stroke while activating the BK channel. This effect is accomplished by promoting the phagocytosis of microglia and can be reversed by Paxilline.

Clearance of apoptotic cell debris is vital to the improvement of stroke outcomes (Yew et al., 2019). Several studies suggest that inhibiting microglial activation results in improved neurogenesis (Hoehn et al., 2005; Liu et al., 2007). Four-week minocycline treatment at 4 days after tMCAO decreased the number of activated microglia and increased the number of BrdU^+^/NeuN^+^ cells (Liu et al., 2007). Overexpressing miR-98 inhibited microglial phagocytosis to attenuate neuronal death in the penumbra area at 3 days after tMCAO (Yang et al., 2021). However, microglial phagocytotic receptor TREM2 knockout mice showed less tissue resorption and a larger lesion area, along with severe limb bias, which suggested that microglial phagocytosis improved synapse and axon regeneration after injury (Werneburg et al., 2020). Neuronal and synapse regeneration contributes to brain neural network remodeling, which improved cognitive and motor function after injury (Wu et al., 2020). Our evidence supported the notion that activation of BK channels promoted microglial phagocytosis *in vitro* and *in vivo* and alleviated neuronal loss at 3 day after stroke, which was accompanied by better neurobehavioral score and less co-location of TUNEL and NeuN.

The phenotype of microglia changes dynamically during several disease progression, therefore exhibiting different effects on injury (Ma et al., 2017). In the acute stage of ischemic stroke, microglia tend to polarize into M2 (Zhao et al., 2017), which participated in clearance of dead cells and release of anti-inflammatory factors (Wang et al., 2018), meanwhile reducing the level of pro-inflammatory factors (Lambertsen et al., 2019). Our results showed that the regulation of BK channels had no impact on microglial polarization, which explained why there was no significant difference of the inflammatory factor levels among DMSO, NS19504, and Paxilline groups (Figure 6A).

Extracellular signal-regulated kinase 1/2 (ERK1/2) is a key member of the mitogen-activated protein kinase (MAPK) family, which can be stimulated by cellular growth factors. Activated ERK1 and ERK2 are transferred into the nucleus and activate downstream transcription factors and modulate cell proliferation and differentiation (Zhang and Liu, 2002). In the nervous system, ERK1/2 participates in the neuroprotection effect of several components. Plasmalogens (PIs) alleviated neuronal apoptosis after serum deprivation via activating the MAPK/ERK pathway (Sun et al., 2015). The brain-derived neurotrophic factor (BDNF) stimulated the differentiation and survival of human umbilical cord blood mesenchymal stem cells (HUCB-MSCS) into neurons (Lim et al., 2008) through the activation of MAPK/ERK. Inhibition of ERK promoted HMGB1-mediated neuronal death *in vitro* (Kim et al., 2011). ERK1/2 was involved in promoting neuronal survival and differentiation (Tejeda and Diaz-Guerra, 2017; Xie et al., 2020). After ischemic stroke, cell debris of injury induce microglial phagocytosis *via* the TREM-2/DAP12/ERK/PKC pathway (Fu et al., 2014). TREM2 participates in microglial phagocytosis of apoptotic neurons (Wu et al., 2017) and displays anti-inflammation effects after cerebral ischemic and reperfusion injury (Takahashi et al., 2005). Stimulation of TREM2 on microglia increased ERK/MAPK phosphorylation and promoted microglial phagocytosis, which was reversed after ERK inhibitor treatment (Takahashi et al., 2005). Our results supported that significantly upregulated p-ERK1/2 protein levels after BK activation were accompanied by the increase of microglial phagocytosis, which suggests that ERK1/2 is involved in the neuroprotection of BK channel-mediated microglial phagocytosis.

Our results found that mice of the NS19504 group showed a significantly lower score of the mNSS test, higher average score of the hinging wire test, and decreased number of TUNEL^+^/NeuN^+^ cells in the NS19504 group at 3 days after tMCAO. During 1–14 days after tMCAO, the NS19504 group had decreased mortality compared with the Paxilline group. We speculated that there may be two reasons for the significant neurological improvement only on 3 days after tMCAO. First, the protein level of p-ERK1/2 increased at 3 days after tMCAO, which participated in neuronal survival. Second, mice were treated within 3 days after surgery, which might limit the effectiveness of NS19504. Our results also showed that BK channel expression decreases up to 14 days after tMCAO; the effect of activating BK channels among different stages of ischemic stroke is important to be further explored. In addition, the limitation of our study is the non-specificity of the BK channel activator and inhibitor, *BK*^*flox/flox*^. *CX3CR1-cre* mice need to be used in the future, which might provide direct evidence for BK-mediated microglial phagocytosis.

In summary, our results demonstrate that BK channels are mainly expressed in microglia and neurons. Activation of BK channels promotes microglial phagocytosis both *in vivo* and *in vitro*, which reduces neuronal loss and alleviates neurobehavior deficit; ERK1/2 was involved in this process. BK channel-mediated microglial phagocytosis may be a potential target for stroke therapy.

## Data Availability Statement

The raw data supporting the conclusions of this article will be made available by the authors, without undue reservation.

## Ethics Statement

The animal study was reviewed and approved by the Institutional Animal Care and Committee (IACUC) of Shanghai Jiao Tong University, Shanghai, China.

## Author Contributions

ZZ was responsible for the generation of the project idea. SH and ZZ were responsible for the experiment design. SH was responsible for the most of the experiment operation, analysis of the results, and generation of the manuscript. TC and QS were responsible for animal behavior test, western blot, primary microglia culture. RS was responsible for the mNSS test. YM and HK were responsible for the polish of the manuscript and experiment operation. G-YY, ZZ, and YT supervised the project. All authors contributed to the article and approved the submitted version.

## Conflict of Interest

The authors declare that the research was conducted in the absence of any commercial or financial relationships that could be construed as a potential conflict of interest.
