# Supplementary material for: BK Channel-Mediated Microglial Phagocytosis Alleviates Neurological Deficit After Ischemic Stroke
Source: Front Cell Neurosci. 2021 Jul 1;15:683769. doi: 10.3389/fncel.2021.683769 (PMC8281043; doi:10.3389/fncel.2021.683769)
Supplement: Supplementary file 1 [file Image_1.pdf]

Supplementary Material

1 Supplementary Figure 1

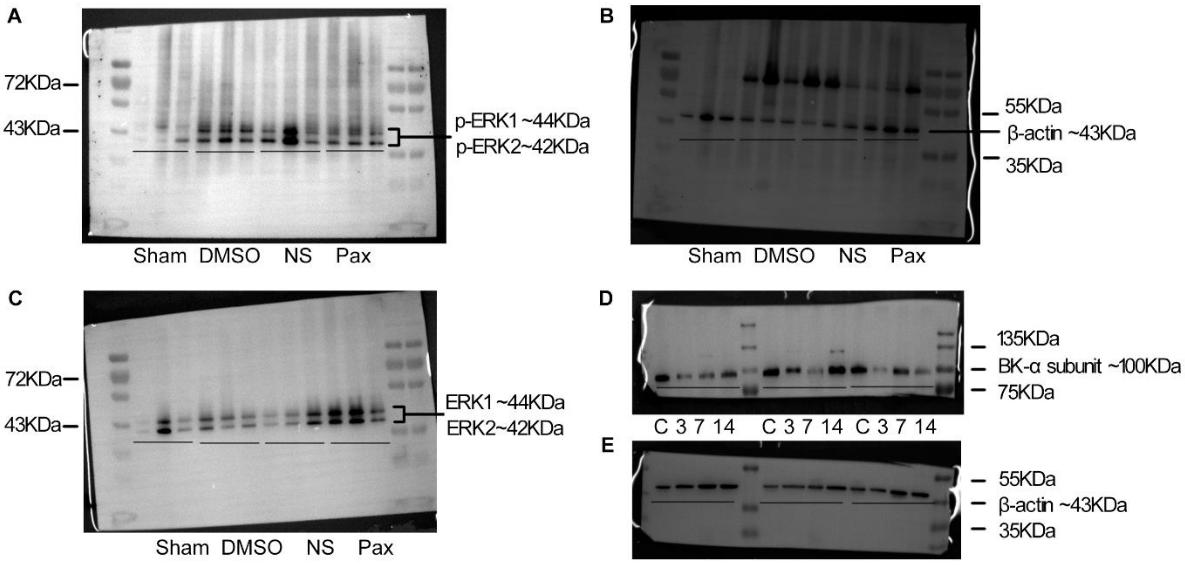

Supplementary Figure 1. This figure contains the full scan of the entire original membrane. **(A)** showed the original membrane of p-ERK1/2 in **Fig.6B**; **(B)** showed the original membrane of β-actin in **Fig.6B**; **(C)** showed the original membrane of t-ERK1/2 in **Fig.6B**, NS, NS19504; Pax, Paxilline; **(D)** showed the original membrane of BK channels in **Fig.1B**; **(E)** showed the original membrane of β-actin in **Fig.1B**, C, Control.
